# The Treatment of Corpulency

**Published:** 1887-12

**Authors:** Ross R. Bunting


### THE TREATMENT OF CORPULENCY.

BY ROSS R. BUNTING, M. D.

The form of obesity commencing at birth, and increasing during infancy and chilhood, is not amenable to treatment, as it is generally fatal before the age of puberty. Where there exists fatty degeneration of the heart, which we frequently find in fat persons, a physician should always be consulted, as the treatment must be modified to suit the particular case. Under forty years of age, *very much* can be accomplished by treatment, although there are some notable exceptions to this rule, as in the case of Dr. Banting, which we will consider farther on. The following are the rules of treatment of obesity without medicine : The quantity of food must be diminished: we must eat less. A medical friend much inclined to corpulence, informed me, that, in his own case, if he reduced the quantity of food, he soon noticed that he became thinner ; when he found he could run to catch a car without getting out of breath, he considered himself all right. Our fat friends must follow the advice given by Abernethy to one of his noble patients : "Your lordship must do as the famous Duke of Wellington did on a well-known occasion,—cut off the supplies, and the enemy will leave the citadel ;" they must take the sort of food that will produce the least amount of fat, but will give strength ; they should therefore take more of nitrogenous and less of carbonaceous food. Of the five different sorts of animal food—beef, veal, mutton, lamb, and pork,—the first is the least, and pork the most heat-producing. The latter, therefore, should not be eaten.

> "Thus saith the Prophet to the Turk :
> Good Mussulman, abstain from pork."

White bread makes heat and fat ; rye is less heating ; corn-bread fattens ; oat-meal is good for strength, not for heat, is very suitable ; rice makes fat ; potato contains a great deal of starch,—not to be eaten ; fruits may be eaten ; milk is too fattening, contains butter and sugar of milk ; coffee and tea can be taken, but not with milk or sugar. Water must be taken very sparingly,—one glassful in the twenty-four hours, if possible. Exercise must not be violent, as that produces an appetite ; yet if it be walking, if perspiration is produced to a slight degree, it is an advantage. Eight hours' sleep is enough, as a rule, and seven will generally be sufficient. Some of this advice is very old, for the *Father of Medicine* says : "These obese persons should eat but one meal a day, should not bathe, should sleep on a hard bed, and should walk as much as possible." Vinegar has been, and is still used as a remedy for obesity, but, taken in sufficient quantity to produce any effect, it is injurious.

There are persons who, from a corpulent condition, become quite thin (which is generally produced by disease), when the skin is in wrinkles ; there appears to be too much skin ; they present a very singular appearance. Those who lose flesh by a systematic treatment do not present this appearance. It is well for a person undergoing treatment to be weighed every two weeks. One pound a week to lose is, in most cases, sufficient. A lady under my care, after an attack of rheumatism, became very stout, weight, 230 pounds; height, five feet six inches. The least exercise produced dyspnœa. At the end of six months, after following the treatment above indicated, her weight was reduced to 190 pounds. She was able to walk without getting out of breath, besides being very comfortable in other ways.

The Banting system, named after Mr. Banting, an undertaker of London, is very much the same as the treatment we have mentioned. This treatment, ordered him by his physician, Harvey, is but a modification of that of Daucel, a French army surgeon, who originated it about forty years since. He was sixty-six years of age, five feet five inches in height; weight, 202 pounds. He says: "I could not stoop to tie my shoe, nor attend to the little offices humanity requires, without considerable pain and difficulty. I have been compelled to go down stairs slowly backward, to save the jar of increased weight upon the ankle and knee-joints, and been obliged to puff and blow with every slight exertion." Under treatment he lost, in little over a year, 46 pounds, and says he "is quite well, and can walk up and down stairs like other men, and can stoop with ease and freedom."

As there are more thin people in the world than fat people, a word of advice to the former might not be out of place. They must sleep all they can; keep early hours for retiring; lie down in the middle of the day; drink a great deal of water; eat heartily, especially of farinaceous food; take plenty of exercise, but in moderation. Be cheerful. Sterne says that "every time a man laughs he adds something to his life." And, according to Solomon, "A merry heart doeth good like a medicine; but a broken spirit drieth the bones." Follow the old adage, "Laugh and grow fat."

